# Stronger Together Toolkit: An Open Resource to Implement Evidence-Based Peer-to-Peer Support Programs for Cancer Patients Globally

**DOI:** 10.1200/GO.22.21000

**Published:** 2022-05-05

**Authors:** Carolyn Taylor, Anna Cabanes, Phuongthao Le, Sarah Afifi

**Affiliations:** ^1^ Global Focus on Cancer, South Salem, NY; ^2^ Johns Hopkins Bloomberg School of Public Health, Baltimore, MD; ^3^ New York University, New York City, NY

## Abstract

The global burden of cancer falls heavily on low-and middle-income countries (LMICs), where there are critical gaps in the provision of supportive care. Initiatives to translate effective, resource-appropriate preventions and clinical treatments in LMICs have been gathering pace, yet few examples exist to guide the translation of interventions that address the high unmet psychosocial needs among cancer patients. To address this gap, we have recently published a theory-based cultural adaptation of this peer mentoring program to the context of Viet Nam (https://pubmed.ncbi.nlm.nih.gov/34365524/). To increase access to evidence-based supportive cancer care, building on our prior work we have developed a toolkit which provides guidance for the implementation of a culturally appropriate and resource adaptable, peer-to-peer support program.

**METHODS:**

A multidisciplinary team of cancer experts and advocates employed a multi-step process to develop a virtual toolkit to guide the implementation of a peer-to-peer support program that coordinates with oncology centers to train and supervise cancer survivor-volunteers to assist cancer patients and their families better understand and navigate their treatment.

**RESULTS:**

The “Stronger Together Toolkit” (STT) is an open-source, self-guided peer support program that allows for the provision of cultural and resource adaptation. STT includes six modules which use resources such as videos, slides, and worksheets, to define the intervention, engage and communicate with stakeholders, assess for readiness and plan for implementation, train program coordinators and peer mentors, evaluate training and implementation, create policies and procedures for different contexts, and identify opportunities for sustainable funding.

**CONCLUSION:**

STT can be used to increase access to supportive cancer care in a way that ensures that the program and activities are acceptable and appropriate to the culture, health system, and resources of the local community and setting. In addition, the STT can be adapted to introduce peer to peer support across additional disease platforms.

